# Future policy and research for advance care planning in dementia: consensus recommendations from an international Delphi panel of the European Association for Palliative Care

**DOI:** 10.1016/S2666-7568(24)00043-6

**Published:** 2024-04-09

**Authors:** Miharu Nakanishi, Sandra Martins Pereira, Lieve Van den Block, Deborah Parker, Karen Harrison-Dening, Paola Di Giulio, Jürgen In der Schmitten, Philip J Larkin, Ninoslav Mimica, Rebecca L Sudore, Iva Holmerová, Ida J Korfage, Jenny T van der Steen

**Affiliations:** Department of Public Health and Primary Care, Leiden University Medical Center, Leiden, Netherlands; Department of Psychiatric Nursing, Tohoku University Graduate School of Medicine, Sendai-shi, Japan; Mental Health Promotion Unit, Research Center for Social Science and Medicine, Tokyo Metropolitan Institute of Medical Science, Tokyo, Japan; CEGE: Research Center in Management and Economics – Ethics and Sustainability Research Area, Católica Porto Business School, Universidade Católica Portuguesa, Porto, Portugal; Vrije Universiteit Brussel-UGent End-of-Life Care Research Group, Vrije Universiteit Brussel, Brussels, Belgium; School of Nursing and Midwifery, Faculty of Health, University of Technology Sydney, Sydney, NSW, Australia; Department of Research and Publications, Dementia UK, London, UK; Faculty of Health and Life Sciences, De Montfort University, Leicester, UK; Department of Public Health and Pediatrics, University of Torino, Torino, Italy; Institute of General Practice/Family Practice, Medical Faculty, University of Duisburg-Essen, Essen, Germany; Institute of Higher Education and Research in Healthcare, Lausanne University Hospital and University of Lausanne, Lausanne, Switzerland; Palliative and Supportive Care Service, Lausanne University Hospital and University of Lausanne, Lausanne, Switzerland; School of Medicine, University of Zagreb, Zagreb, Croatia, Department for Biological Psychiatry and Psychogeriatrics, University Psychiatric Hospital Vrapče, Zagreb, Croatia; Division of Geriatrics, Department of Medicine, University of California, San Francisco, CA, USA; San Francisco Veterans Affairs Medical Center, San Francisco, CA, USA; Centre of Expertise in Longevity and Long-Term Care, Faculty of Humanities, Charles University, Prague, Czech Republic; Department of Public Health, Erasmus University Medical Center, Rotterdam, Netherlands; Department of Public Health and Primary Care, Leiden University Medical Center, Leiden, Netherlands; Radboudumc Alzheimer Center and Department of Primary and Community Care, Radboud university medical center, Nijmegen, Netherlands; Cicely Saunders Institute, Faculty of Nursing, Midwifery and Palliative Care, King’s College London, London, UK

## Abstract

Advance care planning (ACP) is increasingly recognised in the global agenda for dementia care. The European Association for Palliative Care (EAPC) Taskforce on ACP in Dementia aimed to provide recommendations for policy initiatives and future research. We conducted a four-round Delphi study with a 33-country panel of 107 experts between September, 2021, and June, 2022, that was approved by the EAPC Board. Consensus was achieved on 11 recommendations concerning the regulation of advance directives, equity of access, and dementia-inclusive approaches and conversations to express patients’ values. Identified research gaps included the need for an evidence-based dementia-specific practice model that optimises engagement and communication with people with fluctuating and impaired capacity and their families to support decision making, while also empowering people to adjust their decisions if their goals or preferences change over time. Policy gaps included insufficient health services frameworks for dementia-inclusive practice. The results highlight the need for more evidence and policy development that support inclusive ACP practice models.

## Introduction

Living well with dementia is a key element and feature of the global agenda for dementia care.^1^ As a syndrome with several causes that lead to a progressive decline in multiple areas of functioning, dementia has been recognised as a life-limiting condition that benefits from a palliative care approach.^2^ People might have 7–10 years of survival from the onset of dementia,^3^ accompanied by progressive cognitive decline, loss of capacity for decision making, and involving challenges in meaningful communication, physical symptoms, and complex health-care needs.^4^ The number of people with dementia is estimated to increase from 57·4 million globally in 2019, to 152·8 million in 2050, with a larger proportion of this increase in low-income and middle-income countries (LMICs).^5^ The rise would signify an escalating global need for palliative care.^6^ Therefore, dementia should be considered a part of the public health agenda in all countries,^1,7^ with a holistic palliative approach from the point of diagnosis until the end of life.^7,8^ Palliative care constitutes a crucial component of a longevity society.^9^ Specifically, advance care planning (ACP) is recommended if a person with dementia has the capacity to make and express specific decisions, to empower them and improve their quality of care.^8^ ACP also aims to explore, document, and share a person’s preferences about their future care in preparation for when they are no longer able to communicate their wishes.^10,11^ ACP in dementia can promote conversations about dementia-specific illness scenarios, emphasise relational autonomy, and reduce uncertainty about the future.^12–14^ However, ACP is underused in practice among people with dementia.^15–17^ Barriers to ACP implementation might be amplified in dementia, including health-care professionals’ insufficient knowledge about the expected trajectory of dementia and potential medical decisions,^18,19^ concerns regarding the capacity of the person to engage in ACP, low confidence in initiating and having quality conversations,^13,20^ and an absence of perceived benefits to the person with dementia.^21,22^

Policy initiatives might help to address barriers and challenges in implementing ACP in dementia. The literature suggests that the roles and responsibilities of health-care professionals could either facilitate or hinder ACP initiation.^13,23,24^ Because ACP conversations are infrequently initiated by the person with dementia, health-care professionals are recommended to do so instead.^12^ However, they might feel unsure about the best timing to initiate ACP, how to plan for an uncertain future, and how to manage changing decisional capacities and preferences.^18^ Thus, policy initiatives should shape strategic priorities for addressing these barriers and challenges to guide health-care professionals in promoting ACP in dementia. Although national dementia policies have increasingly encompassed a holistic palliative approach^25^ and some of these policies also include ACP in their statements,^26–30^ it is not always mentioned. Furthermore, national dementia policies are not available in all countries, with LMICs^25^ particularly under-represented. In addition, dementia care models have been developed and tested exclusively in high-income countries.^31^ Even though the availability of individual treatments, care, and support services can vary across health-care systems and organisations, planning for future decision making in a given care system is about the person’s important right of access to high-quality dementia care and is fundamental for all, regardless of country of residence. Ensuring ACP is implemented in a way that supports people-centred care is key for policies to achieve equitable access to palliative care.^9^

An international consensus on addressing gaps in policy and research is an important next step to guide dementia policies and future research to optimise ACP in dementia. To address these pressing issues, this study aimed to develop recommendations for policy initiatives to promote ACP in dementia for areas that need further research and to achieve a broad consensus among experts from diverse regions.

## Methods

### Design and setting

We conducted a Delphi study on the basis of the remit of the European Association for Palliative Care (EAPC) Taskforce on ACP in Dementia. The taskforce aimed to conceptualise ACP in dementia in terms of its definition and elements, as published,^14^ and to provide recommendations for practice, policy, and research. The Delphi study had three phases: preparing the conceptualisation and recommendations; recruiting panellists, data collection, and analysis; and the EAPC board of directors’ approval of the report. A framework with a definition and the elements of capacity, family, and engagement and communication specific to dementia achieved consensus.^14^ In this Health Policy, we report on the recommendations for policy and research.

We built upon and expanded the study scope from two previous Delphi studies in palliative care in dementia^2^ and generic ACP.^10^ Delphi studies combine the know-ledge and experience of experts with evidence through a structured iterative process.^32,33^ Consensus among important stakeholders is key to identifying policy and research gaps that cannot be determined by evidence only.

### Ethics procedures

The Medical Research Ethics Committee Leiden Den Haag Delft reviewed the study protocol (reference N21.105) and declared the study exempt from the Dutch Medical Research Involving Human Subjects Act (wet medisch-wetenschappelijk onderzoek met mensen). The study was conducted in accordance with the Declaration of Helsinki. The protocol of the Delphi study was registered at the Open Science Framework^34^ and in the WHO International Clinical Trials Registry Platform (van der Steen, 2021; NL9720), both on Sept 7, 2021, before data collection commenced.

### Participants

A Delphi panel of experts in dementia care and ACP research, practice, and policy were invited for a four to five round online survey. During 9 months, four survey rounds with interim analyses and feedback were conducted between Sept 30, 2021, and June 30, 2022.

We aimed to recruit professionals and researchers as experts, particularly those who were not only knowledgeable, but also end users of the guidance for ACP in dementia in the future. As our study aimed to identify the research and policy gaps, we purposefully identified potential candidates with variations in profession and geographical area. Researchers were included because they would provide an international scientific perspective on ACP. Inclusion criteria were having expertise on ACP, dementia care, or ACP in dementia through practice, policy, research, or clinical experience; sufficient capability to understand, read, and write English; and providing informed consent to participate in the Delphi study as indicated ahead of completing any online survey.

We applied several approaches to sample candidate participants. First, the taskforce of 14 experts^14^ sought potential candidates through their networks, including through their connections and from suggestions from national and international organisations, conferences, and research groups for dementia and palliative care. This diverse taskforce in geographical region and profession^14^ enabled the identification of 102 candidates, yet the majority were in western countries (in Europe, northern America, and Australasia; n=77). Second, we reviewed the list of participants in previous Delphi studies on palliative care in dementia and generic ACP who had already been identified as experts. Third, we listed candidates from dementia-related organisations’ websites. Finally, we searched PubMed by use of the keywords ‘dementia’ and ‘advance care planning’, targeting authors from Africa, Asian countries other than Japan (due to an existing network of researchers in Japan), South America, and central America.

A total of 178 candidates from 46 countries were identified (appendix p 2). On Sept 30, 2021, we invited them to participate in an online survey, aiming to recruit about 100 participants. We based this aim on the numbers and response rates from previous EAPC Delphi studies (64 [72%] of 89 candidates in palliative care in dementia^2^ and 109 [76%] of 144 candidates in generic ACP)^10^ to achieve professional and geographical variation, including those who were under-represented or absent in the previous Delphi studies, such as spiritual counsellors and professionals from non-western countries. Informed consent was provided by ticking a box on the introductory pages in the first round, with a panellist information letter and consent form available for download. Following invitation of the 178 candidates, 169 probably received the invitation in time and 107 experts chose to participate.^14^

### Data collection

Long surveys for the Delphi study were built in Castor EDC Amsterdam—software that enables researchers to easily capture and integrate high-quality data. We sent up to two personal reminders to panellists who agreed to participate but had not completed the survey near the deadline. The same procedure was applied in all four rounds.

### Measurements

Measurements for this study were included in the third and fourth rounds. Participant characteristics were asked in the first round and were reported elsewhere.^14^ Participants were asked to rate their agreement with policy and regulation statements in the round three and four surveys. In round four, the participants were also asked to indicate their priorities of the three issues that deserve particular attention for future research: capacity, family, and engagement and communication. These issues were identified as specific to ACP in people with dementia in the first three rounds.^14^

Policy and regulation statements were developed by the EAPC taskforce.^14^ We revised five statements included in the generic consensus conceptualisation of ACP,^10^ and added two statements. These seven statements were displayed to participants in the third round survey. Another five additions suggested by participants were added and displayed in the fourth round survey. Agreement response options were (with numbers showing the distance and emphasising symmetry) “1: strongly disagree”, “2: moderately disagree”, “3: neither agree nor disagree”, “4: moderately agree”, and “5: strongly agree”.

In addition, in round four, the participants’ priorities of the three dementia-specific issues for future research were ranked. A link to summaries on the three issues was displayed in the survey to enable participants to view and consult it as needed. A response option of “unable to evaluate” was provided. The gaps on research and policy were asked by use of an open question: “what do you believe are the most important gaps regarding ACP in dementia?” Participants were asked to provide comments in three free text boxes (maximum 4196 characters) for gaps on research, policy in the respondent’s country, and policy internationally.

### Analysis

To evaluate consensus for policy and regulation statements, previously developed criteria based on median, dispersion (IQR), and percent agreement were used. Statements that received a median of 5, an IQR less than or equal to 1, and greater than or equal to 80% agreement (scoring 4 or 5) were deemed to have achieved consensus.^2^ If consensus was not achieved in the round three survey (ie, the first survey in this study), we proposed a revised statement and fed back a summary of the comments of the panel, together with the previous rating of the panellist in the invitation to round four email and with a link to the round four survey environment.

A conventional content analysis^35^ was adapted to free text fields for perspectives on the most important gaps in policy and research. Participants’ answers were extracted into a Microsoft Excel file and answers were cut into sections of text that contained a minimum unit of meaning and labelled with a code based on the framework built in the consensus definition of ACP in dementia.^14^ Two researchers (MN and SMP) independently suggested a set of subcategories for the codes. They reviewed the separate lists and discussed to integrate them. Once integrated, two researchers discussed and determined the set of categories based on the previously mentioned framework. Finally, to capture the whole structure of how the experts recognised gaps, categories across research, domestic policy, and policy internationally were reclassified into domains, including dementia-specific practice, health services frameworks, the social aspect, and calls for action on policy and regulation. The reclassification was inspired by the domains in consensus definition of palliative care in dementia^2^ and generic ACP.^10^ All answers were independently analysed and labelled by two researchers (MN [PhD in nursing] and SMP [PhD in bioethics], both with experience of qualitative analyses). Any disagreements were resolved by discussions that were supervised by JTvdS. Because the participants had evaluated ACP definition and elements in earlier rounds,^14^ alignment with the given definition and elements was taken into consideration with the labelling of codes and categories. A post-hoc subgroup analysis was conducted for subgroups of LMICs regarding agreement for policy and regulation statements and research and policy gaps.

## Results

### Participant characteristics

178 individuals from 46 countries were identified as candidates for the Delphi study and were approached, although 9 were not reached as the invitation email was not deliverable (n=8) or not delivered in time (n=1). Of the 169 people invited, 107 candidates from 33 countries participated. The mean age of the panellists was 52·0 years (SD 12·1). 74 self-identified women and 33 men participated. They reported their background as physicians (n=52), physician assistants or nurse practitioners (n=1), nurses (n=21), psychologists (n=12), ethicists (n=9), policy/administration (n=8), social workers (n=5), epidemiologists (n=4), and spiritual counsellors (n=3). Of the 33 countries of residence, 21 were western countries (in Europe, northern America, and Australasia; 88 participants) and 12 were non-western countries (in Asia, the Middle East, Africa, and southern and central Americas; 19 participants). 25 were high-income countries (95 participants) and eight were LMICs (12 participants). More detailed characteristics are reported elsewhere.^14^

### Policy and regulation statements

The seven statements presented in the round three survey included three statements on health policy: regarding decision making as human rights, public awareness, and funding and organisational support. The remaining four statements were about regulation and directives involving advance care directives as legal binding guidance, a format applied for advance care directives, systems to store copies of documentation, and the status of proxy decisions. Six statements immediately achieved consensus (table 1). One statement on the status of proxy decisions required a fourth round: “laws should recognise proxy decision making as legally binding guidance of medical decision making”. Some panellists cautioned against a legal status that is unknown in various jurisdictions. Others pointed out that it was unclear if the decision was always by a proxy designated by the person with dementia. On the basis of these comments, the statement was revised and displayed in the round four survey: “government should determine how input from proxy decision makers is considered in ACP”. Regarding the round three statement, some panellists cautioned against a legal status that is also unknown in various jurisdictions. It was unclear if the decision was always by a proxy designated by the person with dementia. Health-care professionals might question if the proxy decides in the person’s best interest, resulting in dilemmas, and a prerequistite would be a high-quality ACP process. The revised statement did not achieve consensus either. Post-hoc subgroup analysis showed that there was consensus for the original statement in the round three survey within the subgroup of participants from LMICs, as well as from high-income countries. However, there was no consensus for the revised statement in the round four survey within either subgroup (appendix p 4).

The panel suggested additional important recommendations in the round three survey. Thus, five new statements were introduced in the policy and regulation section in round four. These recommendations were for the routine discussions of health-care needs, conceptualisations of ACP, equity of access to any measures stimulating ACP, approaches to ensure optimal value, and encouragement of ACP conversations to discuss and express values. All new statements achieved consensus in the round four survey (table 1). There was also consensus for these statements within the subgroup of participants from LMICs. Among the three dementia-specific issues, engagement and communication received the primary priority ranking, followed by capacity, and then family (appendix p 5).

### Gaps in research, domestic policy, and policy internationally

73 panellists provided their comments on the most important gaps in research and policy. After excluding two answers of “unable to evaluate” and those too short to assign a meaningful code (as judged by the two researchers assigning codes; MN and SMP), a total of 123 answers were analysed. From these answers, 295 codes emerged; codes were classified into 187 subcategories, which were reclassified into 38 categories (table 2; appendix pp 6–16). Finally, the 38 categories were integrated into four domains: dementia-specific practice, health services frameworks, the social aspect of recognition of ACP in dementia, and call for actions on policy and regulation (table 2).

The dementia-specific practice domain had the most codes assigned—24 categories and 79 subcategories— and was considered a research gap more often than a policy gap. Participants requested dementia-specific ACP practice models that are characterised by dementia-specific issues, such as capacity, engagement, preferences, communication, decision-making support with impaired capacity, and family (all categories with ten or more codes). Disease progression accompanied by capacity decline was often noted to potentially affect preferences and decision making in ACP. Engagement and communication were mentioned as specifically challenging in ACP for people with impaired capacity. To address this challenge, a need for decision-making support in ACP conversations was noted. Changes in preferences over time were also highlighted, which would necessitate regular review of ACP as a process. Capacity and change in preferences were noted as the rationales for the need for models of care for communication with patients, decision-making support with people with impaired capacity, and how to interact with family (appendix p 17).

The health services frameworks domain had five categories and 48 subcategories, with frequent mentions of policy gaps both in the participant’s country and internationally (table 2). Participants pointed out an absence of integration of ACP in existing dementia care and health and social care systems. Some of them pinpointed an absence of financial incentives to ACP implementation and others cautioned against misdirection by financial incentives. Participants acknowledged regional and cross-country variation in definitions and acceptance of ACP on the basis of culture and differences in health-care systems, laws, and regulations. Professional training and education were called for to build competency of professionals who interact with people with dementia in front-line caregiving. An implementation strategy was also said to be needed with consideration of feasibility under workforce shortages and competing priorities in health and social care systems. Evaluation in practice was mentioned to monitor the quality of ACP conversations with people with dementia (subcategories in appendix p 18).

The social aspect domain had five categories and 34 subcategories. Participants flagged the gaps equally across research, policy in their own country, and international policy (table 2). Participants referred to social acknowledgment of ACP in dementia as being related to wrong beliefs about capacity and ACP by healthcare professionals. To overcome these wrong beliefs, developing evidence was called for to show the benefits of ACP. Some participants presented their interest in international exchanges to understand cultural diversity in ACP. Nonetheless, other participants pointed out the absence of consensus on what ACP is as the challenge (appendix p 19). Scarce research with patient and public involvement was raised as a research gap by one participant (table 2; appendix p 19).

The call for actions on policy and regulation domain had four categories and 26 subcategories (table 2). Participants reported inadequate policy regarding ACP and legal validation of ACP or a proxy decision maker in their countries, resulting in proxy decision makers not being appointed. Some participants provided an example of ACP and decision making by people with dementia not being legally accredited. Participants cautioned that less attention was paid to ACP in dementia care and little attention was paid to dementia care in the general healthcare context. Cross-country variation in ACP practices was mentioned regarding policy and legal variation. The role of the government was underlined to develop policy and regulations that entitle people with dementia to equal access to ACP (appendix p 20).

Participants from LMICs provided nine answers for research or policy gaps. From the nine answers, a total of 13 codes emerged. Three comments in the dementia-specific practice domain involved categories of engagement and moral dilemmas, but not capacity. There were no mentions regarding the health services frameworks domain. Five comments in the social aspect domain involved categories of social acknowledgment and cultural diversity. Five comments in the call for actions on policy and regulation domain involved the categories of legal validation and policy (appendix p 21).

## Discussion

Based on the input from experts from 33 countries, international consensus for ACP in dementia was obtained for 11 recommendations for policy and regulation. The consensus included health policy regarding decision making as a human right, public awareness, funding and organisational support, advance directive regulation, equity of access, and dementia-inclusive approaches and conversations to express patient values. The research gaps mostly constituted the domain of dementia-specific practice, while the policy gaps involved the domains of health services frameworks, the social aspect, and call for actions on policy and regulation. This work is a crucial step to guide dementia policies and initiatives that address barriers and challenges to implementing ACP in dementia.

The consensus in policy and regulation underscores government actions needed to ensure equity in access to ACP among people with dementia. The actions suggested included formulating procedures, such as regulations on advance directives. Policies to minimise the risk of poor practice were mentioned by the panellists as a tick box practice exercise; professionals would pay most attention to ensure that all requirements for financial incentives were fulfilled or met—little attention would be paid to support people with dementia to discuss and express their values. In addition, national dementia plans often do not have explicit contents of palliative care^25,36^ and palliative care is insufficiently integrated into health-care policies for older people.^37^ Such fragmentation across existing systems could hinder the inclusion of people with dementia in ACP and related practice. Further, the panellists observed little information on ACP being available as a barrier to implementation. ACP context is often absent or presented in legal and medical terms in websites of dementia associations.^38^ People with dementia themselves called for an ACP definition that would be more inclusive for people with dementia, rather than the establishment of dementia-specific ACP.^39^ As the generic EAPC consensus of advance care planning^10^ applies to people with capacity, a dementia-inclusive ACP conceptualisation was needed to see loss of capacity as a gradual and fluctuating process that depends on context and calls for support throughout the dementia trajectory.^39^ In summary, linkage between existing health-care policies across dementia, palliative care, and care for older adults should be strengthened.

One statement on the status of proxy decision makers did not achieve consensus. As acknowledged by participants, proxy decision makers cannot be appointed under some legal systems. Appropriateness of the statement would depend on legal variations across and between countries. Further, some participants questioned whether the proxies decide in the person’s best interest; concordance between the patient’s expressed preferences and proxy care preferences was found to be often suboptimal,^40,41^ with larger discrepancies with more advanced dementia.^42^ Families themselves have information and support needs in decision making on behalf of the person with dementia.^20^ The status of decision making by proxies thus needs further clarification with how to help align decisions with the person’s values and preferences.

Our text analysis denoted a wide range of research and policy gaps in ACP for people with dementia. Dementia-specific practice is frequently mentioned in research gaps with capacity as an outstanding issue, followed by engagement, preferences, communication, decision-making support with impaired capacity, and family. However, within the scope of the three dementia-specific issues,^14^ both capacity (28 codes) and engagement (18 codes) and communication (11 codes) had more mentions than family (10 codes). The primary interest in these first two issues was in alignment with the priority rankings of future research and the issue of family is also involved in surrogate decision making in the context of ACP for people with other diseases.^43,44^ Also, in our Delphi consensus study, only the issue of family needed further rounds of revision and clarification to achieve consensus on family being of importance and specific for dementia.^14^ Therefore, the issues of capacity and engagement and communication might have reflected growing attention, along with an emerging movement to involve the person with dementia in ACP.^20^

Future lack of diminished capacity was often recognised by professionals as a dementia-specific barrier to initiating ACP.^45,46^ Although professionals routinely made judgements about capacity in their daily work, many also had concerns about making a formal assessment that would influence the implementation of a legally binding document.^46^ Experts in our study expressed the need for prudent capacity assessment and evaluation, as suggested by physicians in the USA.^47^ Participants also cautioned that some health-care professionals could underestimate the decisional capacity of people with dementia. Such assumptions could interfere with the ability to engage and communicate with people with dementia,^21^ as professionals have shown gatekeeping behaviours in ACP for them.^48^ Furthermore, the ACP process was deemed to be not straightforward by participants. Their perspectives might have mirrored a shifting ACP model from a clinician-led and documentation-focused process to the broader concept of an ongoing people-centred conversational approach.^20,49–53^ The research gaps we identified highlight the need for evidence that will inform the complexity and ambitious process of the conversation-based model.

The absence of health services frameworks in respondents’ countries was often described as a policy gap. Participants mentioned that some characteristics of the health-care systems in their countries interacted with the dementia-specific issues, resulting in local and national variation in ACP acceptance and processes that challenged implementation in practice. To overcome these barriers to ACP implementation, social aspects and calls for action on policy and regulation were raised in the policy gaps in respondents’ countries. To overcome wrong beliefs and misconceptions among professionals, the panel suggested delivering evidence that would support the benefit of ACP in dementia.

Our subgroup analysis within participants from LMICs revealed some differences in recommendations to fill gaps in research and policy. There were no mentions by participants from LMICs that fell into health services frameworks or capacity issues. The policy gaps were instead mainly perceived as social acknowledgment and legal validation of ACP in dementia. The differences between these issues and those identified in high-income countries could be reflective of a paucity of health and social care systems in LMICs that provide general dementia care, including diagnosis, post-diagnostic support, and ACP. Given the rapid rate of population ageing and requirement for sustainable health-care systems, dementia-inclusive ACP is imperative for LMICs, in alignment with further health policy development.

### Strengths and limitations

The main strength of this study is the diversity of the 33 countries represented among participant experts, including eight LMICs. A combination of quantitative and qualitative analysis provided a comprehensive and in-depth understanding of the needs for policy and research to promote ACP in dementia. A limitation of this work is that our Delphi panel did not seek to include individuals with dementia, as the panellists needed to have expert understanding of evidence and policy to respond to open questions soliciting for suggestions. Further discussion should be sought based on input from people with dementia by use of methods that can reduce choice task complexity, such as discrete choice experiments.^54^ Despite this limitation, the consensus reached, and the identification of research and policy gaps, will provide key starting points for discussion across multiple stakeholders. Future quantitative research to achieve consensus on priorities could be inspired by the qualitative suggestions provided by our diverse panel of experts.

## Conclusion

There was an international consensus on government actions to ensure regulation around ACP, equity in access to ACP for people with dementia, and dementia-inclusive approaches and conversations to express patients’ values. Research gaps were identified on a dementia-specific practice model that optimises engagement and communication with people with impaired capacity and families to support their decision making, while also empowering people to adjust their decisions, should their goals or preferences change over time. Identified policy gaps included existing health services frameworks failing to envision dementia-inclusive practice, which cause substantial variation and challenges in ACP implementation. Policy gaps also included legal validation of ACP and proxy decision making with variation across countries. Guidance by evidence and a call for action on policy and regulation was suggested. The research and policy gaps highlight the need to avoid poor practice by evidence and policy development that will support a dementia-inclusive ACP practice model.

## Supplementary Material

Supplementary appendix

## Figures and Tables

**Table 1:** Final statements for policy and regulation that achieved consensus

	Median	IQR	% agreement	Agreement (n/N)
**Round 3**				
Health policy				
Public awareness should be raised of the importance of ACP for people with dementia, including the aims of ACP, how to engage in ACP, its legal status, and how to access resources to support ACP	5	0	95·2	80/84
Governments, health insurers, and health-care organisations should secure appropriate funding and the organisational support needed for ACP in dementia	5	1	95·2	80/84
Human rights include the right for people with dementia to decide about care, to appoint a proxy decision maker or to participate in shared decision making as preferred, the right to receive support in decision making, and to prevent undue influence as a principle	5	1	90·0	72/80
Regulation around directives				
Governments and health-care organisations need to create reliable and secure systems to store copies of advance care directives (living wills) and documentation of ACP conversations in electronic medical and nursing files if available, so that these are easy to retrieve, transfer, and update	5	0	88·6	62/70
Advance care directives (living wills) need both a structured format to enable easy identification of specific goals and preferences in emergency situations and an open text format so people can describe their values, goals, and preferences, and also a description of specific situations when the directive applies	5	1	93·2	69/74
Laws should recognise advance care directives (living wills) as legally binding guidance of medical decisions about care and treatment the person does not want, if the situation and condition of the person with dementia clearly matches the situation and condition anticipated by the person with dementia at the time the advance care directive was developed	5	1	82·4	61/74
**Round 4**				
Regulation around directives				
Governments and health-care organisations should use conceptualisations of ACP that support living well with dementia from diagnosis until end of life	5	0	96·3	77/80
Governments should advocate routine discussions of health-care needs, particularly in the context of frailty and deterioration	5	1	91·6	76/83
Governments and health-care organisations should use ACP approaches that are targeted and refined enough to ensure optimal value to people with dementia and their families	5	1	91·4	74/81
Governments should ensure equity of access to any measures stimulating ACP that target the general public, aiming at equal benefit for people with dementia and their family of starting conversations outside health-care settings*	5	1	90·0	72/80
Governments and health-care organisations should encourage ACP conversations in which people with dementia are supported to discuss and express their values also if they are not ready to discuss medical issues but wish to discuss, for example, social issues exclusively	5	1	86·4	70/81

Agreement was rated from 1: strongly disagree to 5: strongly agree in the third and fourth round survey. Criteria of achieved consensus included median of 5, IQR less than or equal to 1, and greater than or equal to 80% agreement (score of 4 or 5). There was also a consensus for all statements within the subgroup of participants from low-income and middle-income countries. Five statements were added to the round four survey on the basis of feedback from participants in the round three survey. Not all 107 panellists participated in all four rounds or completed all items. ACP=advance care planning.

* For people with dementia and their families who initiate ACP discussions outside of health-care settings.

**Table 2:** Domains and categories of gaps in research and policy for ACP in dementia

	Total codes	Research codes	Domestic policy codes	International policy codes
**Dementia-specific practice domain**				
Total of categories in domain	139	81	31	27
Capacity	28	13	9	6
Engagement	18	12	4	2
Preferences	13	11	0	2
Communication	11	10	1	0
Decision-making support with impaired capacity	11	7	1	3
Family	10	6	2	2
Prognosis	8	5	1	2
Diagnosis	6	0	3	3
Concordance	5	5	0	0
Continuous conversation model	5	1	3	1
Diversity	4	3	1	0
Initiation or planning ahead	3	3	0	0
Advocacy	3	0	2	1
Opportunity	3	0	2	1
Process	2	2	0	0
Interrelation with depressive symptoms	1	1	0	0
Moral dilemmas	1	1	0	0
Social aspects of life with dementia specifically	1	1	0	0
Appointment of proxy	1	0	1	0
Stakeholders in ACP conversation	1	0	1	0
Consistency	1	0	0	1
Relational approach*	1	0	0	1
Specificity in physician’s practice	1	0	0	1
Communication between different care organisations	1	0	0	1
**Health services frameworks domain**				
Total of categories in domain	69	14	42	13
Health-care system	39	8	20	11
Professional training and education	10	0	8	2
Evaluation	7	4	3	0
Implementation	7	2	5	0
Variation	6	0	6	0
**The social aspect domain**				
Total of categories in domain	46	13	20	13
Social acknowledgment	21	4	11	6
Evidence	11	5	6	0
Cultural diversity	7	3	0	4
Consensus	6	0	3	3
Patient and public involvement in research	1	1	0	0
**Calls for action on policy and regulation domain**				
Total of categories in domain	41	0	31	10
Legal validation	19	0	16	3
Policy	15	0	14	1
Cross-country variation	6	0	0	6
Government	1	0	1	0

Data=n. Codes were assigned to respondents’ answers on gaps in research in research, policy in their country, and policy internationally regarding ACP in dementia. A total of 123 answers yielded 295 codes. The 295 codes were integrated into 187 subcategories, which were integrated into 38 categories. Finally, 38 categories were integrated into four domains. ACP=advance care planning.

* Well established relationships with health-care professionals were essential to engage in ACP.

## Data Availability

Data supporting the study’s findings will be made available on reasonable request after approval of a proposal from the last author (JTvdS) after publication of all three white papers addressing the three research questions. Data collected for the analysis, including de-identified individual participant data and a data dictionary defining each field in the set, will then be made available to others on request. The study protocol, which includes a plan for statistical analysis, is available from the Open Science Framework. The standard informed consent form used in the online survey will also be available on request.
