# Supplementary material for: Future policy and research for advance care planning in dementia: consensus recommendations from an international Delphi panel of the European Association for Palliative Care
Source: Lancet Healthy Longev. Author manuscript; Available in PMC 2024 Jul 22. (PMC11262782; doi:10.1016/S2666-7568(24)00043-6)
Supplement: Supplementary appendix [file NIHMS2004167-supplement-Supplementary_appendix.pdf]

THE LANCET
Healthy Longevity

Supplementary appendix

This appendix formed part of the original submission and has been peer reviewed. We post it as supplied by the authors.

Supplement to: Nakanishi M, Martins Pereira S, Van den Block L, et al. Future policy and research for advance care planning in dementia: consensus recommendations from an international Delphi panel of the European Association for Palliative Care. *Lancet Healthy Longev* 2024; published online April 9. https://doi.org/10.1016/S2666-7568(24)00043-6.

Supplementary material

Contents

- Supplementary Table 1. Source of identification and characteristics of potential candidates (n=169) – page 2
- Supplementary Table 2. Policy and regulation statement that did not achieve a consensus – page 4
- Supplementary Table 3. Priority ranking of the three dementia-specific issues for advance care planning (ACP) in persons with dementia for future research – page 5
- Supplementary Table 4. Domains, categories and detail on sub-categories that emerged in most important gaps in research, policy in respondent's country, and policy internationally regarding advance care planning (ACP) in dementia – page 6
- Supplementary Panel 1. Quotations of comments representing six outstanding sub-categories in the 'Dementia-specific practice' domain – page 17
- Supplementary Panel 2. Quotations of comments representing five sub-categories in the 'Health services frameworks' domain – page 18
- Supplementary Panel 3. Quotations of comments representing five sub-categories in the 'The social aspect' domain – page 19
- Supplementary Panel 4. Quotations of comments representing four sub-categories in the 'Call for actions on policy and regulation' domain – page 20
- Supplementary Table 5. Sub-group analysis: Number of codes from low- and middle-income countries (LMICs) in domains and categories of most important gaps in research, policy in respondent's country, and policy internationally regarding advance care planning (ACP) in dementia – page 21

Supplementary Table 1. Source of identification and characteristics of potential candidates (n=169)

	Group	N
Source of identification (more possible)	Networks of EAPC ACP in dementia taskforce (connections and via-via suggestions from national and international organisations, conferences and research groups for dementia and palliative care)	101
	Participants in previous Delphi study	42
	Palliative care in dementia	24
	Generic ACP	18
	Dementia-related organisations' websites	18
	PubMed search using keywords of 'dementia' and 'advance care planning' with targeting authors from Africa, Asia other than Japan, and the South and Central Americas	24
Profession (more possible)	Physician, physician assistant or nurse practitioner	79
	Nurse (any level)	36
	Psychologist	22
	Ethicist	10
	Policy/administration	27
	Social worker	8
	Epidemiologist (self-report by respondents)	4
	Spiritual counsellor	3
	Other, including epidemiologist, sociologist, occupational therapist, lawyer, economist, gerontologist, philosopher, speech-language therapist, physiotherapist and pharmacist	18
Country classification by continent	Western country: Europe, Northern Americas, Australasia and alternating between continents	129
	Non-western country: Asia, Middle East, Africa, Southern and Middle Americas	40
Country classification by income	HIC	141
	LMIC	28

Legend. ACP, advance care planning. EAPC, European Association for Palliative Care. HIC, high income country. LMIC, low- and middle-income country. A total of 169 candidates from 43 countries; of 178 candidates from 46 countries invited, 9 were excluded as invitation email not deliverable (n=8) or did not receive invitation in time (n=1). Multiple sources of identification and multiple professions could apply for one candidate: the sum of numbers is not equal to the total number of candidates. Profession of respondents (n=107) is based on self-report in the survey, while that of non-respondents (n=62) was based on publicly available information used to aim at recruiting a diverse sample. The response rate was significantly lower among candidates from non-western countries (47·5%) compared with those from western countries (68·2%; $\chi^2(1)=5\cdot64$, P=·018). Similarly, the response rate of candidates from LMICs (42·9%) was significantly lower than those from HICs (67·4%;

$\chi^2(1)=6{\cdot}05$, $P={\cdot}014$). Caution: given that candidates from non-western countries and LMICs were invited more often after identifying them through internet searches rather than through personal connections, which is generally a less effective recruitment strategy, we cannot interpret the significantly different response rates in terms of response bias.

Supplementary Table 2. Policy and regulation statement that did not achieve a consensus

Original statement (round 3 survey)	Group	Median	IQR	% of agreement	N agreement / total
Laws should recognize proxy decision making as legally binding guidance of medical decision making	Total (no consensus)	4	1·5	75·0	54/72
	High-income countries (no consensus)	4	2	72·7	48/66
	Low- and middle-income countries (consensus)	5	1	100·0	6/6
**Revised statement (round 4 survey)**		**Median**	**IQR**	**%**	
Government should determine how input from proxy decision makers is considered in ACP	Total (no consensus)	4	2	74·1	63/85
	High-income countries (no consensus)	4	1·5	75·0	60/80
	Low- and middle-income countries (no consensus)	4	2	60·0	3/5

Legend. ACP, advance care planning. In the original statement, some panellists cautioned against a legal status which is also unknown in various jurisdictions. It was unclear if the decision was always by a proxy designated by the person with dementia. Healthcare professionals may question whether the proxy decides in the person's best interest resulting in dilemmas, and a prerequisite would be a high-quality ACP process in place.

Supplementary Table 3. Priority ranking of the three dementia-specific issues for advance care planning (ACP) in persons with dementia for future research

	**N of responses**		
**Dementia-specific issues**	**1st**	**2nd**	**3rd**
Engagement and communication	38	25	12
Capacity	26	23	26
Family	13	27	35

Legend. ACP, advance care planning. Participants in the round 4 survey were asked to indicate how they prioritize the three issues (capacity, family, and engagement and communication) for future research, which were identified as specific for ACP in persons with dementia in round 1 and 2 surveys. Out of 107 participants, 11 declined ticking the box "unable to evaluate". Another 21 did not provide any answer. The remaining 75 participants provided their priority rankings of the three issues.

Supplementary Table 4. Domains, categories and detail on sub-categories that emerged in most important gaps in research, policy in respondent's country, and policy internationally regarding advance care planning (ACP) in dementia

Domain	Category	Sub-category	N of codes			
			Total	Research	Policy	
					Country	Internationally
Dementia-specific practice	Total of category	*Total of category*	139	81	31	27
	Capacity	*Total of sub-category*	28	13	9	6
		Assessment of capacity	7	5	1	1
		Underestimated capacity	6	0	3	3
		Impact of disease progression on decision making	3	3	0	0
		Decision making in impaired capacity	2	2	0	0
		Evidence of impact of disease progression on decision making	2	0	1	1
		Implications for the evolution of the disease	1	1	0	0
		Nature of capacity decline over time	1	1	0	0
		Prognosis of capacity	1	1	0	0
		Advanced decision while a person had capacity	1	0	1	0
		Assessment of change in capacity	1	0	1	0
		Concerns about moral dilemmas	1	0	1	0
		Obligation of ACP to people with impaired capacity	1	0	1	0
		Supported ACP to maximize residual capacity	1	0	0	1
	Engagement	*Total of sub-category*	18	12	4	2
		Patient and family engagement	7	7	0	0
		Patient engagement	4	2	0	2

	Patient involvement	2	2	0	0
	Question against patient engagement	2	0	2	0
	Family involvement	1	1	0	0
	Approach to maximize patient engagement	1	0	1	0
	Early engagement	1	0	1	0
Preferences	*Total of sub-category*	13	11	0	2
	Change in preferences	6	6	0	0
	Assessment of wishes and preferences	2	1	0	1
	Domains and thoughts that the person find relevant to planning ahead	1	1	0	0
	Gap between precedent preferences and medical decision making at the moment	1	1	0	0
	Shared preferences between service providers and at care transition	1	1	0	0
	Values that best guide patient, family, and providers	1	1	0	0
	Estimated change in preferences vs ACP	1	0	0	1
Communication	*Total of sub-category*	11	10	1	0
	Serious illness communication	6	6	0	0
	Communication tailored to people with impaired capacity	3	3	0	0
	Communication	1	1	0	0
	Open communication	1	0	1	0
Decision making support with impaired capacity	*Total of sub-category*	11	7	1	3
	ACP adaptation for people with impaired capacity	5	5	0	0
	Supported ACP to engage people with impaired capacity	3	0	0	3
	Question against ACP adaptation	1	1	0	0
	Shared decision making	1	1	0	0

	Tools to engage in high quality decision making	1	0	1	0
Family	*Total of sub-category*	10	6	2	2
	Interaction between patient and family	2	2	0	0
	Opposing argument against family	2	0	1	1
	Bereaved family	1	1	0	0
	Domains and thoughts that family find relevant to planning ahead	1	1	0	0
	Family dynamics in decision making	1	1	0	0
	Risks of focusing on family	1	1	0	0
	Attention paid to caring family in policy	1	0	0	1
	Relying on family about care quality management	1	0	1	0
Prognosis	*Total of sub-category*	8	5	1	2
	Hypothetical situations to be discussed	2	2	0	0
	Nature of disease progression	2	2	0	0
	Evidence of nature of disease progression	2	0	1	1
	Physical treatment options	1	1	0	0
	Impact of progression of dementia on ACP	1	0	0	1
Diagnosis	*Total of sub-category*	6	0	3	3
	Early and correct diagnosis	3	0	2	1
	Disclosure of dementia diagnosis	2	0	1	1
	Timely diagnosis	1	0	0	1
Concordance	*Total of sub-category*	5	5	0	0
	Risk factors for incongruence between ACP and actual care received	3	3	0	0
	Approach to ensure care in alignment with ACP	1	1	0	0
	Goal concordant care	1	1	0	0

Continuous conversation model	*Total of sub-category*	5	1	3	1
	Regular review of ACP	3	0	2	1
	Process of change and monitoring engagement	1	1	0	0
	Concern about tick box exercise	1	0	1	0
Diversity	*Total of sub-category*	4	3	1	0
	Diversity issues	2	1	1	0
	Differences in outcome by types of dementia	1	1	0	0
	Variety of stages of dementia	1	1	0	0
Initiation or planning ahead	*Total of sub-category*	3	3	0	0
	Timing to initiate	2	2	0	0
	Guidance to initiate	1	1	0	0
Advocacy	*Total of sub-category*	3	0	2	1
	Assessment of best interest	1	0	1	0
	Balance between advocacy and autonomy	1	0	1	0
	Risk of institutionalizing ACP	1	0	0	1
Opportunity	*Total of sub-category*	3	0	2	1
	Early ACP encouragement	2	0	1	1
	Opportunity to discuss	1	0	1	0
Process	*Total of sub-category*	2	2	0	0
	Definition of ACP process	1	1	0	0
	Longitudinal process	1	1	0	0
Interrelations with	*Total of sub-category*	1	1	0	0

	depressive symptoms					
		Impact of depression on preferences and decisions	1	1	0	0
	Moral dilemmas	*Total of sub-category*	1	1	0	0
		Moral dilemmas and ethical consideration	1	1	0	0
	Social aspects of life with dementia specifically	*Total of sub-category*	1	1	0	0
		Planning for social aspects of life with dementia	1	1	0	0
	Appointment of proxy	*Total of sub-category*	1	0	1	0
		Consistency in appointment of a proxy decision maker	1	0	1	0
	Stakeholders in ACP conversation	*Total of sub-category*	1	0	1	0
		Identifying stakeholders in ACP	1	0	1	0
	Consistency	*Total of sub-category*	1	0	0	1
		Consistency of approach	1	0	0	1
	Relational approach	*Total of sub-category*	1	0	0	1
		Lack of well-established relationship with healthcare professionals	1	0	0	1
	Specificity in physician's practice	*Total of sub-category*	1	0	0	1
		Fitness to physician's practice	1	0	0	1
	Communication between different care organizations	*Total of sub-category*	1	0	0	1
		Communication between different care organizations	1	0	0	1
Health services frameworks	Total of category	*Total of category*	69	14	42	13

Healthcare system	*Total of sub-category*	39	8	20	11
	Financial incentives in public healthcare system	8	0	6	2
	Lack of information in provider	2	0	2	0
	Lack of information in user	2	0	2	0
	Timely access to health and social care	2	0	1	1
	Timely access to palliative care	2	0	1	1
	Concern about financial incentives	1	1	0	0
	Dementia-friendly healthcare approach	1	1	0	0
	Digital innovation	1	1	0	0
	How to promote ACP in medical settings	1	1	0	0
	Post-diagnostic support	1	1	0	0
	Regulations on ACP	1	1	0	0
	Relationship with existing palliative care system	1	1	0	0
	Translation of expressed wishes in medical decision making	1	1	0	0
	Dementia not eligible for palliative care	1	0	1	0
	Financial disincentives in public healthcare system	1	0	1	0
	Information sharing policy for proxy	1	0	1	0
	Integration into existing healthcare approach	1	0	1	0
	Integration into existing palliative care system	1	0	1	0
	Misdirection by financial incentives in public healthcare system	1	0	1	0
	Palliative care is under-presented	1	0	1	0
	Resources granted by the government	1	0	1	0
	Access	1	0	0	1
	Integration into post-diagnostic support services	1	0	0	1

	Laws on euthanasia	1	0	0	1
	Overcoming procedural perspective	1	0	0	1
	Pharmacological interventions	1	0	0	1
	Service provision	1	0	0	1
	Transparency in medical records	1	0	0	1
Professional training and education	*Total of sub-category*	10	0	8	2
	Competency in workforce	5	0	4	1
	Professional education and training is needed	5	0	4	1
Evaluation	*Total of sub-category*	7	4	3	0
	Outcome measures in ACP process	1	1	0	0
	Outcomes	1	1	0	0
	Quality of ACP conversations	1	1	0	0
	Quality of life	1	1	0	0
	Quality indicator definition	1	0	1	0
	Quality indicator monitoring	1	0	1	0
	Quality measures	1	0	1	0
Implementation	*Total of sub-category*	7	2	5	0
	Implementation guidelines	2	0	2	0
	Feasibility with workforce shortages and competing priorities	1	1	0	0
	Sustainable implementation	1	1	0	0
	Implementation strategies	1	0	1	0
	Support for ACP implementation	1	0	1	0
	Support for providers in ACP implementation	1	0	1	0

	Variation	*Total of sub-category*	6	0	6	0
		Regional variation in laws and regulations	2	0	2	0
		Lack of consistency across different policies	1	0	1	0
		Regional variation in healthcare systems	1	0	1	0
		Regional variation in terminology	1	0	1	0
		Variation in electronic patient medical record systems	1	0	1	0
The social aspect	Total of category	*Total of category*	46	13	20	13
	Social acknowledgement	*Total of sub-category*	21	4	11	6
		Palliative care in dementia is underrecognized	3	0	2	1
		Misbelief as no benefit in healthcare professionals	2	1	1	0
		Awareness raising	2	0	2	0
		Stigma	2	0	1	1
		Cultural difference in understanding and continuity	1	1	0	0
		Misbelief about capacity in healthcare professionals	1	1	0	0
		Misbelief about care and support in healthcare professionals	1	1	0	0
		Approach to general public	1	0	1	0
		Cultural change	1	0	1	0
		Dementia not a healthcare priority	1	0	1	0
		Dementia-friendly cities	1	0	1	0
		Prior information	1	0	1	0
		Attention paid to caring patient in policy	1	0	0	1
		Less attention to ACP in dementia	1	0	0	1
		Open mind	1	0	0	1
		Person centredness	1	0	0	1

	Evidence	*Total of sub-category*	11	5	6	0
		Benefit of ACP in dementia	4	4	0	0
		Data unavailable around end-of-life care	2	0	2	0
		Data unavailable around quality of care	2	0	2	0
		Trial on ACP with family	1	1	0	0
		Evidence in cost-effectiveness of ACP	1	0	1	0
		Lack of information in policy makers	1	0	1	0
	Cultural diversity	*Total of sub-category*	7	3	0	4
		Cultural adaptation	2	1	0	1
		Cultural difference and awareness	1	1	0	0
		Cultural variation	1	1	0	0
		Cultural acceptance of ACP	1	0	0	1
		Culturally defined variation	1	0	0	1
		Variation in central value and belief	1	0	0	1
	Consensus	*Total of sub-category*	6	0	3	3
		Consensus in what ACP is	2	0	1	1
		Consensus in healthcare professionals' role	1	0	1	0
		Definition in disease management	1	0	1	0
		Family role	1	0	0	1
		Patient role	1	0	0	1
	Public Patient Involvement in research	*Total of sub-category*	1	1	0	0
		Patient involvement in research	1	1	0	0
Call for actions on	Total of category	*Total of category*	41	0	31	10

policy and regulation						
	Legal validation	*Total of sub-category*	19	0	16	3
		Power of attorney in advance to capacity loss	4	0	2	2
		Legal status of ACP	3	0	3	0
		Legal validity of ACP in people with dementia	3	0	3	0
		Proxy decision making is not allowed	3	0	3	0
		Family cannot be a proxy decision maker	1	0	1	0
		Integration into existing legal system	1	0	1	0
		Legal status in healthcare professionals' role	1	0	1	0
		Legal status of advance directives	1	0	1	0
		Legislation on medical attorney	1	0	1	0
		National laws	1	0	0	1
	Policy	*Total of sub-category*	15	0	14	1
		No policy regarding ACP	5	0	5	0
		Integration into existing dementia policy	2	0	2	0
		Integration into existing national dementia strategy	2	0	2	0
		Attention paid to case management and in-home care settings in policy	1	0	1	0
		Attention paid to earlier phases of dementia in policy	1	0	1	0
		Attention paid to quality of care in policy	1	0	1	0
		Question about role of policy	1	0	1	0
		Recommendation for ACP	1	0	1	0
		Implementation policy	1	0	0	1
	Cross-country variation	*Total of sub-category*	6	0	0	6

	Diversity in policy	1	0	0	1
	Diversity in recommendation	1	0	0	1
	Legal validation and outcome	1	0	0	1
	Range of legally binding	1	0	0	1
	Variation in dementia care	1	0	0	1
	Variation in quality of ACP	1	0	0	1
Government	*Total of sub-category*	1	0	1	0
	Role of the government	1	0	1	0

Legend. ACP, advance care planning. From 123 answers, 295 codes were yielded. The 295 codes were integrated into 187 sub-categories, and 187 sub-categories were re-classified into 38 categories. Finally, 38 categories were integrated into four domains.

Supplementary Panel 1. Quotations of comments representing six outstanding sub-categories in the 'Dementia-specific practice' domain

Capacity

- *'The minute people hear dementia they jump to capacity. Given non capacity is presumed more often than not I think the priority really needs to be one that supports inclusion for as long as possible [...] What innovations can be offered to support better communication around ACP and improved participation of persons with dementia?' (No.22, North America)*

Engagement

- *'The continuing issue of how to optimally engage patients and families in an ongoing process. How to optimally involve patients who are losing capacity in ongoing advance care planning' (No.8, North America)*

Preferences

- *'How family and healthcare professional reach consensus on when to enact preferences made in ACP when a patient has lost the capacity and their current preferences appear in conflict with their precedent ones outlined in ACP? especially morally difficult decisions such to withdraw/withhold life-sustaining treatments' (No.29, South Pacific)*

Communication

- *'Moreover, a specific communication process should be developed among geriatricians on how to inform and communicate diagnosis, dementia evolution, and the decisions (not only at the end of life) which will affect dementia trajectory' (No.37, Southern Europe)*

Decision making support with impaired capacity

- *'I also think it would be interesting to think about supported ACP as we think about supported decision making. Can someone with impaired capacity still be supported in engaging in ACP and how?' (No.22, North America)*

Family

- *'Hidden agendas within families that drive treatment decisions/demands in supported decision-making' (No.136, South-West Pacific)*

Supplementary Panel 2. Quotations of comments representing five sub-categories in the 'Health services frameworks' domain

Healthcare system

- *'Dementia is still not treated as a terminal disorder. People are often not eligible for palliative care services because of this' (No.21, North America)*
- *'Financial regulations for GP's to attend multidisciplinary meetings and have ACP related conversations' (No.139, Western Europe)*
- *'In fact, they are often monetarily rewarded because when the patient returns to the nursing home, their care is covered by [public benefit scheme in the respondent's country], before reverting back to the lower reimbursement from [another benefit scheme] or private pay' (No.7, North America)*

Professional training and education

- *'Many people with dementia live in care homes/institutions. There is little training or capacity of the care workers to be involved in ACP even though they are closely involved in day to day life of residents' (No.176, North America)*

Evaluation

- *'Evaluating quality of ACP discussions or conversations. Defining the parameters that are important for dementia-specific ACP discussions' (No.101, South-East Asia)*

Implementation

- *'Ways to implement ACP for persons with various stages of dementia, in the real world with health and aged care staff shortages and competing priorities' (No.57, South-West Pacific)*

Variation

- *'Lack of standardization particularly about proxy decision-makers. Laws vary widely by states and health care systems interpretation of existing laws also varies' (No.137, North America)*

Supplementary Panel 3. Quotations of comments representing five sub-categories in the 'The social aspect' domain

Social acknowledgement

- *'Especially with a movement afoot in palliative care in the [respondent's country]. by some leaders in the field to say that ACP does not work and we need to move on from it (I disagree strongly with this)' (No.10, North America)*
- *'There is a public prejudice that decision-making is not possible with severe dementia or even immediately after diagnosis. The idea that we have to provide support to people with dementia is an abandonment of assessment and an attempt to avoid the individualized approach that is the basic concept of the ACP' (No.30, East Asia)*

Evidence

- *'The cost-effectiveness evidence of ACP that ensure the policy change in governments (most of them are utilitarianism) is still lacking' (No.31, East Asia)*

Cultural diversity

- *'Most research on ACP in dementia is conducted in European/American/Australian countries in which there is a bigger aspect of individual choice & values in deciding one's preferred care. There needs to be more research done in Southeast Asian countries to understand better how ACP should be tailored according to the society values' (No.156, South-East Asia)*

Consensus

- *'Divergent understanding what APC is' (No.67, Central Europe)*

Public Patient Involvement in research

- *'In my experience the most important gap is the attitude to involve PWD in research, related to a kind of dichotomic vision (able/unable)' (No.85, Southern Europe)*

Supplementary Panel 4. Quotations of comments representing four sub-categories in the 'Call for actions on policy and regulation' domain

Legal validation

- *'According to [the respondent's country] law, the family involvement in decision making on behalf of patient is not allowed and the person is not allowed to indicate a healthcare proxy' (No.1, Central Europe)*
- *'The residual capacity of decision-making in people with dementia on various issues is not well recognised by clinicians, laws, and of course general public in consequence. Following the first point, the legal validity of ACP is often challenged by family members, clinicians, and judges when the assumed situations in ACP actually happen' (No.31, East Asia)*

Policy

- *'Little or not mention of palliative and end of life care in national dementia strategy. Emphasis is given to timely diagnosis, pharmacological interventions, both very important, but providing high-quality care is very neglected' (No.44, North-western Europe)*
- *'ACP policy generally has been oriented towards European-centric countries with established health & long term care system. There is not yet a law in place to make someone a legal enduring power of attorney (EPOA) when a person (who is expected to lose capacity) hasn't lose their capacity. This means one cannot choose and make someone else their legal EPOA before they got to the point that they lose capacity' (No.156, East Asia)*

Cross-country variation

- *'It is hard to compare ACP internationally because the legal status of documents like living will and advanced directives varies even within the country. It would be interesting to compare the impact/implications of the difference in legal status between countries. [...] I am curious as to whether that results in more/less invasive treatments to prolong life occurring etc' (No.21, North America)*

Government

- *'The role of government' (No.172, East Asia)*

Supplementary Table 5. Sub-group analysis: Number of codes from low- and middle-income countries (LMICs) in domains and categories of most important gaps in research, policy in respondent's country, and policy internationally regarding advance care planning (ACP) in dementia

Domain	Category	N of codes			
		Total	Research	Policy	
				Country	Internationally
Dementia-specific practice	*Total of category*	3	3	0	0
	Engagement	2	2	0	0
	Moral dilemmas	1	1	0	0
Health services frameworks	*Total of category*	0	0	0	0
The social aspect	*Total of category*	5	1	2	2
	Social acknowledgement	3	0	2	1
	Cultural diversity	2	1	0	1
Call for actions on policy and regulation	*Total of category*	5	0	3	2
	Legal validation	4	0	2	2
	Policy	1	0	1	0

Legend. From nine answers, 13 codes were yielded. The following categories did not have any mentions by participants from low- and middle-income countries. Dementia-specific practice: Capacity, Preferences, Communication, Decision making support with impaired capacity, Family, Prognosis, Diagnosis, Concordance, Continuous conversation model, Diversity, Initiation or planning ahead, Advocacy, Opportunity, Process, Interrelations with depressive symptoms, Social aspects of life with dementia specifically, Stakeholders in ACP conversation, Relational approach, Specificity in physician's practice, Communication between different care organizations. Health services frameworks: Healthcare system, professional training and education, Evaluation, Implementation, Variation (all categories). The social aspect: Evidence, Consensus, Public Patient Involvement in research. Call for actions on policy and regulation: Cross-country variation, Government.
